# Is Artificial Intelligence Better Than Human Clinicians in Predicting Patient Outcomes?

**DOI:** 10.2196/19918

**Published:** 2020-08-26

**Authors:** Joon Lee

**Affiliations:** 1 Data Intelligence for Health Lab Cumming School of Medicine University of Calgary Calgary, AB Canada; 2 Department of Community Health Sciences Cumming School of Medicine University of Calgary Calgary, AB Canada; 3 Department of Cardiac Sciences Cumming School of Medicine University of Calgary Calgary, AB Canada

**Keywords:** patient outcome prediction, artificial intelligence, machine learning, human-generated predictions, human-AI symbiosis

## Abstract

In contrast with medical imaging diagnostics powered by artificial intelligence (AI), in which deep learning has led to breakthroughs in recent years, patient outcome prediction poses an inherently challenging problem because it focuses on events that have not yet occurred. Interestingly, the performance of machine learning–based patient outcome prediction models has rarely been compared with that of human clinicians in the literature. Human intuition and insight may be sources of underused predictive information that AI will not be able to identify in electronic data. Both human and AI predictions should be investigated together with the aim of achieving a human-AI symbiosis that synergistically and complementarily combines AI with the predictive abilities of clinicians.

## Absence of Human-Generated Predictions in Patient Outcome Research

In recent years, there has been a proliferation of patient outcome prediction research that applies machine learning (ML) and artificial intelligence (AI) to electronic health records (EHRs) and other clinical and administrative health data. The central premises are that 1) complex health data contains predictive information that ML can effectively extract and transform into a predictive algorithm and 2) accurate prediction of patient outcomes can facilitate early, preventative intervention and more efficient health care resource allocation through identification of high-risk patients. For example, predicting which intensive care unit patients are likely to develop sepsis can prompt early initiation of fluid resuscitation, vasopressor therapy, or antibiotics, which can reduce damage from insufficient organ perfusion [1,2]. Although AI has been enormously successful in medical imaging diagnostics, where the medical condition of interest is already present or absent in the images (eg, diagnosis of diabetic retinopathy [3] and classification of skin legions [4]), patient outcome prediction poses an inherent challenge of predicting events that have *not* *yet* occurred (eg, mortality, length of stay, and readmission) [5]. This challenge is common to both AI and human clinicians.

Interestingly, while human and AI predictions are often directly compared in medical imaging research [6-8], patient outcome prediction studies tend to focus only on ML and seldom investigate human predictions. This is corroborated by a number of systematic reviews and meta-analyses, which target only ML methods [9-14] or empirical methods [15-19]. This gap in the literature is coherent across a wide range of medical specialties and diseases, including trauma [9], cancer [11], neurosurgery [10], depression [12], acute gastrointestinal bleeding [13], sepsis [14], acute liver failure [15], ischemic stroke [16], thermal injury [17], and cardiovascular disease [18,19]. The absence of human predictions appears to be a recent trend, as older literature prior to the current widespread use of modern ML and EHRs includes more comparisons of human and AI predictions [20-23].

There are several possible reasons why human performance is more frequently studied in medical imaging than in patient outcome prediction. First, radiologists are trained to analyze, interpret, and classify images, whereas most other medical specialists are not trained to directly predict patient outcomes. While accurate prognostic information can certainly be helpful in any medical specialty, it is usually generated by empirical risk scoring systems such as the Framingham Risk Score [24] or Acute Physiology and Chronic Health Evaluation (APACHE) [25] rather than by human clinicians. Second, human predictions in medical imaging are readily available from routine clinical practice or can be generated systematically by trained radiologists. Conversely, it is rare for clinicians in other medical specialties to record patient outcome predictions that *they* generate on a regular basis. Third, the implicit assumption is that humans cannot accurately predict patient outcomes because analysis of complex, high-dimensional clinical data may be required; moreover, recall bias is rampant in the human mind.

## Humans and AI Should Work as a Team

However, there is no reason to rule out the possibility that human clinicians can outperform AI in patient outcome prediction, at least in some clinical scenarios. While AI can only access information that can be recorded in the form of electronic data, human clinicians interact face-to-face with their patients and have access to both clinical and contextual information. The qualitative information collected via clinicians’ five senses can be critical in patient outcome prediction; however, this information is mostly absent in EHRs, if it is possible to record it at all. Although some qualitative observations can be recorded in EHRs as free-text notes, such as nursing notes, these data are logged in a limited, inconsistent fashion. Human intuition and insight may well be the most underused resources in patient outcome prediction.

While the performance of ML-based patient outcome prediction models appears impressive on paper, the most accurately predicted cases tend to be “easy” cases where the likely outcomes are already obvious to human clinicians [26]. This further supports the hypothesis that human clinicians perform well in patient outcome prediction.

On the other hand, AI easily outperforms humans in processing, analyzing, and finding patterns in complex, high-dimensional data [27]. As demonstrated by IBM Watson [28] and AlphaGo [29], the memory, attention, and information processing abilities of AI vastly exceed the capabilities of human cognition [30]. This AI advantage is crucial for extracting and using data-driven insights from big data [31]; it is also key to the recent successful breakthroughs in ML, particularly in deep learning [32], in a number of problem domains, including medical imaging [33]. In addition, AI does not suffer from fatigue [34] or cognitive biases (eg, recall bias) [35] as humans do. However, even if AI outperforms human clinicians in patient outcome prediction, human performance represents a more meaningful benchmark that puts AI performance in better perspective. Understanding the superiority of AI in comparison with humans can facilitate adoption of AI technology in real patient care.

The bottom line is that both AI and humans can make unique contributions to patient outcome prediction, and they should help each other to maximize predictive performance. Patient outcome prediction research should aim for human-AI symbiosis, where the respective predictive abilities of AI and human clinicians are combined in a synergistic and complementary way [36]. Given the challenging nature of patient outcome prediction, creating an AI to act alone without human help will simply lead to suboptimal predictive performance because even state-of-the-art ML technology cannot leverage information that is not present in the data [26].

Another way for AI and humans to work together is via the human-in-the-loop model, where humans directly inform machines on how to learn from the data at hand by providing guidance based on human intuition and knowledge. The term “interactive machine learning” [37] was coined to describe this paradigm; it encompasses more well-known branches of ML, such as active learning, where humans select which data points should be labelled. This human-in-the-loop approach can greatly reduce the computational complexity of some ML problems; for example, it has shown promising results in protein folding [38]. Moreover, in the field of human-computer interaction, the human-in-the-loop concept has been studied in the context of vehicle control [39], security [40,41], and decision-making [40,42]. Knowledge from these application areas can potentially inform the design of human-AI symbiosis in patient outcome prediction.

AI and human prediction performance may vary across different types of patients. Complex patterns in data can be more predictive than human intuition in certain patient subgroups, and the opposite may be true in other subpopulations. An investigation of how AI and human predictions can be optimally combined for different types of patients could directly contribute to advancing precision medicine. A better understanding of the respective predictive powers of AI and humans in various clinical scenarios can also help increase human trust in AI (eg, “For this type of patient, I need to trust AI more because most predictive information is buried in the complex data”). This can facilitate evidence-based adoption of AI technology.

For human clinicians to completely trust AI, it is necessary to understand *why* an algorithm arrives at a given conclusion; this requires transparency, traceability, and causality. The active field of explainable AI has been producing useful methods, such as SHapley Additive exPlanations (SHAP) [43], that can help explain how ML models work at an algorithmic level (this explanation is almost always based on correlation rather than causation); however, human clinicians ultimately want to elevate this algorithmic explainability to a model that is understandable by humans with sufficient causal understanding, also known as causability [44]. Therefore, mapping explainability to causability will be key in achieving true human-AI symbiosis.

One major roadblock to the proposed human-AI symbiosis is the need to collect a large number of human predictions in a variety of clinical scenarios, which is labor-intensive and adds to clinicians’ workloads. Seamlessly integrated electronic prediction collection platforms (eg, embedded in a multi-center EHR system) can minimize this burden and enable large-scale prediction collection.

## From Patient Outcome Prediction to Real Impact

Once predictive performance is optimized via human-AI symbiosis, the next important step is to formulate clinical guidelines so that the predictive information is actionable. This is a crucial step, as accurate predictions alone will not lead to any real impact; rather, the combination of accurate predictions and appropriate interventions by clinicians will have a greater effect [5,26].

The ultimate goal of patient outcome prediction is to improve patient outcomes and decrease health care costs through early intervention and efficient use of health care resources. To prove that this goal has been met, we will need to perform randomized clinical trials of AI-driven patient care [45], such as that conducted by Wijnberge and colleagues [46]. In addition to simply comparing AI with human work alone, these randomized clinical trials should investigate a promising third species: human-AI symbiosis.

## Abbreviations

AI: artificial intelligence
APACHE: Acute Physiology And Chronic Health Evaluation
EHR: electronic health record
ML: machine learning
SHAP: SHapley Additive exPlanations

## References

[1] Desautels T, Calvert J, Hoffman J, Jay M, Kerem Y, Shieh L, Shimabukuro D, Chettipally U, Feldman M, Barton C, Wales D, Das R (2016). Prediction of Sepsis in the Intensive Care Unit With Minimal Electronic Health Record Data: A Machine Learning Approach. JMIR Med Inform.

[2] Lee J, Mark RG (2010). An investigation of patterns in hemodynamic data indicative of impending hypotension in intensive care. Biomed Eng Online.

[3] Gulshan V, Peng L, Coram M, Stumpe MC, Wu D, Narayanaswamy A, Venugopalan S, Widner K, Madams T, Cuadros J, Kim R, Raman R, Nelson PC, Mega JL, Webster DR (2016). Development and Validation of a Deep Learning Algorithm for Detection of Diabetic Retinopathy in Retinal Fundus Photographs. JAMA.

[4] Esteva A, Kuprel B, Novoa RA, Ko J, Swetter SM, Blau HM, Thrun S (2017). Dermatologist-level classification of skin cancer with deep neural networks. Nature.

[5] Lindsell CJ, Stead WW, Johnson KB (2020). Action-Informed Artificial Intelligence-Matching the Algorithm to the Problem. JAMA.

[6] Liu X, Faes L, Kale AU, Wagner SK, Fu DJ, Bruynseels A, Mahendiran T, Moraes G, Shamdas M, Kern C, Ledsam JR, Schmid MK, Balaskas K, Topol EJ, Bachmann LM, Keane PA, Denniston AK (2019). A comparison of deep learning performance against health-care professionals in detecting diseases from medical imaging: a systematic review and meta-analysis. Lancet Digit Health.

[7] Emblem KE, Nedregaard B, Hald JK, Nome T, Due-Tonnessen P, Bjornerud A (2009). Automatic glioma characterization from dynamic susceptibility contrast imaging: brain tumor segmentation using knowledge-based fuzzy clustering. J Magn Reson Imaging.

[8] Emblem KE, Pinho MC, Zöllner FG, Due-Tonnessen P, Hald JK, Schad LR, Meling TR, Rapalino O, Bjornerud A (2015). A generic support vector machine model for preoperative glioma survival associations. Radiology.

[9] Liu NT, Salinas J (2017). Machine Learning for Predicting Outcomes in Trauma. Shock.

[10] Senders JT, Staples PC, Karhade AV, Zaki MM, Gormley WB, Broekman ML, Smith TR, Arnaout O (2018). Machine Learning and Neurosurgical Outcome Prediction: A Systematic Review. World Neurosurg.

[11] Kourou K, Exarchos TP, Exarchos KP, Karamouzis MV, Fotiadis DI (2015). Machine learning applications in cancer prognosis and prediction. Comput Struct Biotechnol J.

[12] Lee Y, Ragguett R, Mansur R, Boutilier J, Rosenblat J, Trevizol A, Brietzke E, Lin K, Pan Z, Subramaniapillai M, Chan T, Fus D, Park C, Musial N, Zuckerman H, Chen V, Ho R, Rong C, McIntyre R (2018). Applications of machine learning algorithms to predict therapeutic outcomes in depression: A meta-analysis and systematic review. J Affect Disord.

[13] Shung D, Simonov M, Gentry M, Au B, Laine L (2019). Machine Learning to Predict Outcomes in Patients with Acute Gastrointestinal Bleeding: A Systematic Review. Dig Dis Sci.

[14] Islam M, Nasrin T, Walther B, Wu C, Yang H, Li Y (2019). Prediction of sepsis patients using machine learning approach: A meta-analysis. Comput Methods Programs Biomed.

[15] Wlodzimirow KA, Eslami S, Chamuleau RAFM, Nieuwoudt M, Abu-Hanna A (2012). Prediction of poor outcome in patients with acute liver failure-systematic review of prediction models. PLoS One.

[16] Fahey M, Crayton E, Wolfe C, Douiri A (2018). Clinical prediction models for mortality and functional outcome following ischemic stroke: A systematic review and meta-analysis. PLoS One.

[17] Hussain A, Choukairi F, Dunn K (2013). Predicting survival in thermal injury: a systematic review of methodology of composite prediction models. Burns.

[18] van Dieren S, Beulens J, Kengne A, Peelen L, Rutten G, Woodward M, van der Schouw DSY, Moons K (2012). Prediction models for the risk of cardiovascular disease in patients with type 2 diabetes: a systematic review. Heart.

[19] Damen JAAG, Hooft L, Schuit E, Debray TPA, Collins GS, Tzoulaki I, Lassale CM, Siontis GCM, Chiocchia V, Roberts C, Schlüssel MM, Gerry S, Black JA, Heus P, van der Schouw YT, Peelen LM, Moons KGM (2016). Prediction models for cardiovascular disease risk in the general population: systematic review. BMJ.

[20] Grove W, Zald D, Lebow B, Snitz B, Nelson C (2000). Clinical versus mechanical prediction: A meta-analysis. Psychol Assess.

[21] Gardner W, Lidz CW, Mulvey EP, Shaw EC (1996). Clinical versus actuarial predictions of violence in patients with mental illnesses. J Consult Clin Psychol.

[22] Marchese MC (1992). Clinical versus actuarial prediction: a review of the literature. Percept Mot Skills.

[23] Bandiera G, Stiell IG, Wells GA, Clement C, De Maio V, Vandemheen KL, Greenberg GH, Lesiuk H, Brison R, Cass D, Dreyer J, Eisenhauer MA, MacPhail I, McKnight R, Morrison L, Reardon M, Schull M, Worthington J (2003). The Canadian C-Spine rule performs better than unstructured physician judgment. Ann Emerg Med.

[24] D'Agostino RB, Vasan RS, Pencina MJ, Wolf PA, Cobain M, Massaro JM, Kannel WB (2008). General cardiovascular risk profile for use in primary care: the Framingham Heart Study. Circulation.

[25] Zimmerman JE, Kramer AA, McNair DS, Malila FM (2006). Acute Physiology and Chronic Health Evaluation (APACHE) IV: Hospital mortality assessment for today’s critically ill patients*. Crit Care Med.

[26] Chen JH, Asch SM (2017). Machine Learning and Prediction in Medicine - Beyond the Peak of Inflated Expectations. N Engl J Med.

[27] Jarrahi MH (2018). Artificial intelligence and the future of work: Human-AI symbiosis in organizational decision making. Bus Horiz.

[28] Ferrucci D, Brown E, Chu-Carroll J, Fan J, Gondek D, Kalyanpur A, Lally A, Murdock J, Nyberg E, Prager J, Schlaefer N, Welty C (2010). Building Watson: An Overview of the DeepQA Project. AIMag.

[29] Silver D, Huang A, Maddison CJ, Guez A, Sifre L, van den Driessche G, Schrittwieser J, Antonoglou I, Panneershelvam V, Lanctot M, Dieleman S, Grewe D, Nham J, Kalchbrenner N, Sutskever I, Lillicrap T, Leach M, Kavukcuoglu K, Graepel T, Hassabis D (2016). Mastering the game of Go with deep neural networks and tree search. Nature.

[30] Grigsby SS (2018). Artificial intelligence for advanced human-machine symbiosis. Augmented Cognition: Intelligent Technologies. AC 2018. Lecture Notes in Computer Science.

[31] L'Heureux A, Grolinger K, Elyamany H, Capretz M (2017). Machine Learning With Big Data: Challenges and Approaches. IEEE Access.

[32] LeCun Y, Bengio Y, Hinton G (2015). Deep learning. Nature.

[33] Martín Noguerol T, Paulano-Godino F, Martín-Valdivia MT, Menias CO, Luna A (2019). Strengths, Weaknesses, Opportunities, and Threats Analysis of Artificial Intelligence and Machine Learning Applications in Radiology. J Am Coll Radiol.

[34] Hockey G, Wiethoff M (1993). Cognitive fatigue in complex decision-making. Adv Space Biol Med.

[35] Dawson NV, Arkes HR (1987). Systematic errors in medical decision making:. J Gen Intern Med.

[36] Topol EJ (2019). High-performance medicine: the convergence of human and artificial intelligence. Nat Med.

[37] Holzinger A (2016). Interactive machine learning for health informatics: when do we need the human-in-the-loop?. Brain Inform.

[38] Cooper S, Khatib F, Treuille A, Barbero J, Lee J, Beenen M, Leaver-Fay A, Baker D, Popović Z, Players Foldit (2010). Predicting protein structures with a multiplayer online game. Nature.

[39] Driggs-Campbell K, Shia V, Bajcsy R (2015). Improved driver modeling for human-in-the-loop vehicular control. 2015 IEEE International Conference on Robotics and Automation (ICRA).

[40] Schumann MA, Drusinsky D, Michael JB, Wijesekera D (2014). Modeling Human-in-the-Loop Security Analysis and Decision-Making Processes. IIEEE Trans. Software Eng.

[41] Cranor LF (2008). A Framework for Reasoning About the Human in the Loop. usenix.org.

[42] Subramania HS, Khare VR (2011). Pattern classification driven enhancements for human-in-the-loop decision support systems. Decision Support Systems.

[43] Lundberg SM, Erion G, Chen H, DeGrave A, Prutkin JM, Nair B, Katz R, Himmelfarb J, Bansal N, Lee S (2020). From Local Explanations to Global Understanding with Explainable AI for Trees. Nat Mach Intell.

[44] Holzinger A, Langs G, Denk H, Zatloukal K, Müller H (2019). Causability and explainability of artificial intelligence in medicine. Wiley Interdiscip Rev Data Min Knowl Discov.

[45] Angus DC (2020). Randomized Clinical Trials of Artificial Intelligence. JAMA.

[46] Wijnberge M, Geerts B, Hol L, Lemmers N, Mulder M, Berge P, Schenk J, Terwindt L, Hollmann M, Vlaar A, Veelo D (2020). Effect of a Machine Learning-Derived Early Warning System for Intraoperative Hypotension vs Standard Care on Depth and Duration of Intraoperative Hypotension During Elective Noncardiac Surgery: The HYPE Randomized Clinical Trial. JAMA.

